# Supplementary material for: The mechanosensitive Piezo1 channel is required for bone formation
Source: eLife. 2019 Jul 10;8:e47454. doi: 10.7554/eLife.47454 (PMC6685704; doi:10.7554/eLife.47454)
Supplement: Supplementary file 1. [file elife-47454-supp1.docx]

**Supplementary File 1. Clinical features of fracture patients involved in bone specimens analysis.**

| **Non-osteoporosis** | **Age** | **Gender** | **T score for BMD at Spine** | **BMD at**  **Femoral neck** | **Diagnosis** | **PINP**  **(ng/ml)** | **β-CTX**  **(ng/ml)** |
| --- | --- | --- | --- | --- | --- | --- | --- |
| 1 | 81 | woman | 0.6 | 0.729 | Left femoral neck fracture | 33.29 | 0.62 |
| 2 | 85 | woman | 0.2 | 0.699 | Left femoral neck fracture | 26.07 | 0.23 |
| 3 | 79 | woman | 0.2 | 0.643 | Left femoral neck fracture | 43.67 | 0.56 |
| 4 | 73 | woman | -0.2 | 0.619 | Left femoral neck fracture | 94.77 | 0.96 |
| 5 | 68 | woman | -0.3 | 0.624 | Left femoral neck fracture | 30.02 | 0.44 |
| 6 | 84 | woman | -0.5 | 0.723 | Left femoral neck fracture | 168.4 | 0.97 |
| 7 | 74 | woman | -0.8 | 0.638 | Right femoral neck fracture | 22.98 | 0.27 |
| 8 | 79 | woman | -1.3 | 0.68 | Left femoral neck fracture | 73.58 | 0.92 |
| 9 | 85 | woman | -1.4 | 0.586 | Right femoral neck fracture | 40.15 | 0.53 |
| 10 | 83 | woman | -1.5 | 0.78 | Right femoral neck fracture | 77.91 | 0.67 |
| **Osteoporosis** | **Age** | **Gender** | **T score for BMD at Spine** |  | **Diagnosis** | **PINP**  **(ng/ml)** | **β-CTX**  **(ng/ml)** |
| 1 | 68 | woman | -2.5 | 0.694 | Left femoral neck fracture | 28.65 | 0.92 |
| 2 | 74 | woman | -2.6 | 0.729 | Left femoral neck fracture | 36.21 | 0.51 |
| 3 | 83 | woman | -2.7 | 0.71 | Left femoral neck fracture | 43.74 | 0.78 |
| 4 | 75 | woman | -2.7 | 0.631 | Left femoral neck fracture | 47.82 | 0.97 |
| 5 | 71 | woman | -2.8 | 0.613 | Left femoral neck fracture | 38.7 | 0.84 |
| 6 | 89 | woman | -2.9 | 0.507 | Right femoral neck fracture | 32.95 | 0.62 |
| 7 | 85 | woman | -2.9 | 0.629 | Right femoral neck fracture | 32.95 | 0.62 |
| 8 | 73 | woman | -3.0 | 0.688 | Right femoral neck fracture | 79.45 | 0.96 |
| 9 | 68 | woman | -3.7 | 0.749 | Left femoral neck fracture | 45.28 | 1.37 |
| 10 | 81 | woman | -4.1 | 0.524 | Right femoral neck fracture | 63.13 | 0.55 |

The patients were recruited at between 65 and 90 years of age. The classification of the patients in the osteoporotic and the non-osteoporotic group was based on DXA evaluation. The T-score were measured for BMD at spine of women. Biochemical analysis was performed according to manufacturer’s protocol. Serum procollagen type I C propeptide (PICP) and type I collagen (β-CTX) were measured by ELISA.
