# Supplementary material for: The mechanosensitive Piezo1 channel is required for bone formation
Source: eLife. 2019 Jul 10;8:e47454. doi: 10.7554/eLife.47454 (PMC6685704; doi:10.7554/eLife.47454)
Supplement: Supplementary file 2. [file elife-47454-supp2.docx]

**Supplementary File 2 mRNA Primers are used as follows**

**Mouse primers**

| *Gapdh*-Forward | 5’-AACATCAAATGGGGTGAGGCC-3’ |
| --- | --- |
| *Gapdh*-Reverse | 5’-GTTGTCATGGATGACCTTGGC-3’ |
| *Alp*-Forward | 5’-ATCTTTGGTCTGGCTCCCATG-3’ |
| *Alp*-Reverse | 5’-TTTCCCGTTCACCGTCCAC-3 |
| *Bglap*-Forward | 5’-CCAAGCAGGAGGGCAATA-3’ |
| *Bglap*-Reverse | 5’-TCGTCACAAGCAGGGTCA-3’ |
| *Col1α1*-Forward | 5’-GGGACCAGGAGGACCAGGAAGT-3’ |
| *Col1α1*-Reverse | 5’-GGAGGGCGAGTGCTGTGCTTT-3’ |
| *Piezo1*-Forward | 5’-TCATCATCCTTAACCCATGGTG-3’ |
| *Piezo1*-Reverse | 5’-TGAAGACGATAGCTGTCATCCA-3’ |
| *Dmp1*-Forward | 5’-CTGAAGAGAGGACGGGTGATT-3’ |
| *Dmp1*-Reverse | 5’-CGTGTGGTCACTATTTGCCTG-3’ |
| *Sost*-Forward | 5’-AGCCTTCAGGAATGATGCCAC-3’ |
| *Sost*-Reverse | 5’-CTTTGGCGTCATAGGGATGGT-3’ |

**Human primers**

| *GAPDH*-Forward | 5’-ACAACTTTGGTATCGTGGAAGG-3’ |
| --- | --- |
| *GAPDH*-Reverse | 5’-GCCATCACGCCACAGTTTC-3’ |
| *ALP*-Forward | 5’-GTGAACCGCAACTGGTACTC-3’ |
| *ALP*-Reverse | 5’-GAGCTGCGTAGCGATGTCC-3’ |
| *BGLAP*-Forward | 5’-GGCGCTACCTGTATCAATGG-3’ |
| *BGLAP*-Reverse | 5’-GTGGTCAGCCAACTCGTCA-3’ |
| *COL1A1*-Forward | 5’-GAGGGCCAAGACGAAGACATC-3’ |
| *COL1A1*-Reverse | 5’-CAGATCACGTCATCGCACAAC-3’ |
| *PIEZO1*-Forward | 5’-CAATGAGGAGGCCGACTACC-3’ |
| *PIEZO1*-Reverse | 5’-GCACTCCTGCAGTTCGATGA-3’ |
| *CTSK*-Forward | 5’-AAGCCAGACAACAGATTTCCAT-3’ |
| *CTSK*-Reverse | 5’-GGATCATTTGAAGCACAAACAA-3’ |
| *ACP5*-Forward | 5’-GGAGGGAATAAAGGCTCAGG-3’ |
| *ACP5*-Forward | 5’-GGAACTCAGCAAAGGTGAGC-3’ |
| *MMP9*-Forward | 5’-CGTCGGTCCGTCCGCTA-3’ |
| *MMP9*-Reverse | 5’-TCAGCCCTCACCTCGGTACT-3’ |
| *DMP1*-Forward | 5’-AGGAAGTCTCGCATCTCAGAG-3’ |
| *DMP1*-Reverse | 5’-TGGAGTTGCTGTTTTCTGTAGAG-3’ |
| *SOST*-Forward | 5’-ACACAGCCTTCCGTGTAGTG-3’ |
| *SOST*-Reverse | 5’-GGTTCATGGTCTTGTTGTTCTCC-3’ |

The sequence of primers were used for detecting mRNA expression by quantitative PCR.
